# Age at menopause and lifetime cognition: Findings from a British birth cohort study

**DOI:** 10.1212/WNL.0000000000005486

**Published:** 2018-05-08

**Authors:** Diana Kuh, Rachel Cooper, Adam Moore, Marcus Richards, Rebecca Hardy

**Affiliations:** From the MRC Unit for Lifelong Health and Ageing at UCL, London, UK.

## Abstract

**Objective:**

We investigated whether cognitive performance between ages 43 and 69 years was associated with timing of menopause, controlling for hormone replacement therapy, childhood cognitive ability, and sociobehavioral factors.

**Methods:**

We used data from 1,315 women participating in the Medical Research Council National Survey of Health and Development (a British birth cohort study) with known age at period cessation and up to 4 assessments of verbal memory (word-learning task) and processing speed (letter-cancellation task) at ages 43, 53, 60–64, and 69. We fitted multilevel models with linear and quadratic age terms, stratified by natural or surgical menopause, and adjusted for hormone replacement therapy, body mass index, smoking, occupational class, education, and childhood cognitive ability.

**Results:**

Verbal memory increased with later age at natural menopause (0.17 words per year, 95% confidence interval [CI]: 0.07–0.27, *p* = 0.001); an association remained, albeit attenuated, after full adjustment (0.09, 95% CI: 0.02–0.17, *p* = 0.013). Verbal memory also increased with later age at surgical menopause (0.16, 95% CI: 0.06–0.27, *p* = 0.002), but this association was fully attenuated after adjustment. Search speed was not associated with age at menopause.

**Conclusion:**

Our findings suggest lifelong hormonal processes, not just short-term fluctuations during the menopause transition, may be associated with verbal memory, consistent with evidence from a variety of neurobiological studies; mechanisms are likely to involve estrogen receptor β function. Further follow-up is required to assess fully the clinical significance of these associations.

A recent review of 13 observational studies showed that later age at menopause and longer reproductive life are generally associated with better cognitive function or delayed cognitive decline. However, a call was made for the evidence to be strengthened by using prospective studies with larger, age-homogeneous samples, longer follow-up periods, repeated cognitive assessments, and adjustment for a wider range of sociobehavioral factors associated with timing or type of menopause and cognition.^1,2^

Using data from birth cohort studies, we and others have shown that higher prior cognitive ability, assessed as early as childhood, is associated with later age at natural menopause,^3–6^ hysterectomy,^7^ and later cognitive function,^8,9^ and attenuated cross-sectional associations between menopausal status and cognition.^10^ Lifelong studies are required given this evidence, and the broader evidence from animal and human studies showing pleiotropic effects of estrogen across life on the central and peripheral nervous systems, reproductive, and other body systems.^11–13^

In a British birth cohort study, we investigated whether verbal memory and processing speed, assessed 4 times between ages 43 and 69, were associated with timing of period cessation due to natural or surgical menopause. The study controlled for hormone replacement therapy (HRT) use, body size, adult socioeconomic factors, smoking, education, and childhood cognitive ability. We hypothesized that later natural or surgical menopause would be associated with better cognition but would be explained by childhood cognitive ability and other covariables.

## Methods

The Medical Research Council (MRC) National Survey of Health and Development (NSHD) is a sample of all births in 1 week in March 1946 in mainland Britain comprising 5,362 (2,547 female) individuals followed up 24 times, so far to age 69, with a further 9 postal questionnaires to women during midlife.^14^ The maximum sample for these analyses comprised 1,315 women with information on age at period cessation and at least one adult cognitive assessment. Of the original birth cohort, 288 (11.3%) had already died, 246 (9.7%) were living abroad, 403 (15.8%) had been lost to follow-up, and 295 (11.6%) had not provided all necessary data.

### Standard protocol approvals, registrations, and patient consents

Ethical approval for the most recent visit was given by Queen Square Research Ethics Committee (13/LO/1073) and Scotland A Research Ethics Committee (14/SS/1009). Participants provided written informed consent for each visit.

### Adult cognition

Trained nurses assessed verbal memory at 43, 53, 60–64, and 69 years using a 15-item word-learning task repeated 3 times giving a maximum possible score of 45. Two sets of word lists were alternated over waves to minimize practice effects. They also assessed processing speed using a visual search task requiring participants to cross out as quickly and accurately as possible, in 1 minute, the letters P and W randomly embedded in a grid of other letters; the maximum score was 600.^4,8^

### Type and timing of menopause

We obtained information on menstrual irregularity, month and year of last menstrual cycle, any operation to remove the uterus or ovaries (validated against hospital records), and monthly HRT use from the home visits and annual postal questionnaires between ages 47 and 54 (inclusive) and at age 57. We calculated months since birth until periods ceased naturally, or because of bilateral oophorectomy (with or without hysterectomy), or because of hysterectomy with or without unilateral oophorectomy. We excluded women whose periods stopped for other reasons (n = 37), such as chemotherapy. We also excluded 122 women starting HRT before menopause who had not ceased HRT for at least a year from the main analyses because it was not possible to assign a date of menopause; however, they contributed to additional HRT analyses.

### Other covariables

We chose covariables based on the scientific literature, including previous NSHD studies that have shown that childhood cognitive ability, education, body size, smoking, and occupational class were associated with cognitive scores,^8,15–17^ and related to type or timing of period cessation.^3,4,18–20^ From reported monthly histories of HRT, we derived “ever use” (yes vs no), use at the time of the cognitive tests, years of use, and years since last use. We derived body mass index (BMI) from height (m) and weight (kg) measured according to standard protocols at each nurse visit. We categorized the participant's own current or most recent occupation into 6 classes (from professional to unskilled manual) according to the Registrar General's social classification. In addition, we distinguished those who reported smoking at least one cigarette a day at each of the nurse visits from lifelong nonsmokers and ex-smokers. We classified highest educational qualifications attained by age 26 into the following: degree or higher; advanced secondary qualifications, usually attained at age 18; ordinary secondary qualifications, usually obtained at age 16; lower level qualifications; and none. We derived a standardized measure of childhood cognitive ability from tests of reading comprehension, pronunciation, vocabulary, and nonverbal reasoning at age 8, chosen because previous NSHD research had shown that the association between childhood cognitive ability and timing of menopause was strongest at this age.^3,4^

### Main analyses

We used Stata version 14.2 (StataCorp, College Station, TX) for all analyses. For each cognitive test, we fitted multilevel models that account for the correlation of repeated cognitive scores within individuals. In preliminary analyses for verbal memory including linear and quadratic terms for age, there was evidence of an interaction between type of menopause and both age terms (*p* < 0.05), so we performed all analyses separately for natural and surgical menopause. We modeled change in verbal memory and processing speed by linear and quadratic age terms, and fitted intercept and slope as random effects.

We first included age at period cessation and its interaction with each age term to test whether changes in cognitive scores varied by age at period cessation. We then performed a series of adjustments: for HRT use as a time-varying covariable; additionally for BMI and smoking as time-varying covariables, educational qualifications and own occupational class; and additionally for childhood cognitive ability. We added each covariable with any relevant age interactions. In the model for women who had surgical menopause, we tested whether the associations of bilateral oophorectomy were different from the associations of hysterectomy with conservation of at least one ovary, and whether the effect of age at surgery or HRT on verbal memory was modified by type of procedure.

### Additional analyses

We compared the mean and SDs of each cognitive score at each age in the maximum available samples by age at period cessation and type of menopause, then stratified by menopause type and repeated the age at period cessation analyses (tables e-1 to e-5, links.lww.com/WNL/A428). We used linear regression models to test these relationships. To check that the results were not influenced by morbidity associated with surgery performed for cancers, we reran the multilevel models excluding those cases. Considering some published evidence that associations with surgical menopause may be weaker with increasing age at time of procedure, we then reran the models excluding women who had a surgical menopause after age 50.^21^ We also checked whether additionally adjusting for parity affected the results. Because the sample with known age at period cessation did not include all women who took HRT, we ran linear regression models, stratified by type of menopause, to examine the associations between verbal memory and processing speed at age 69 with ever use, duration, and years since last use of HRT. We adjusted for covariables and additionally adjusted for the same cognitive test at age 43 to test whether HRT use was associated with rate of decline.

### Data availability

Data are available on request to the NSHD Data Sharing Committee. NSHD data sharing policies and processes meet the requirements and expectations of the UK MRC policy on sharing of data from population and patient cohorts: mrc.ac.uk/publications/browse/mrc-policy-and-guidance-on-sharing-of-research-data-from-population-and-patient-studies/. Data requests should be submitted to mrclha.swiftinfo@ucl.ac.uk; further details can be found at nshd.mrc.ac.uk/data.aspx. These policies and processes are in place to ensure that the use of data from this national birth cohort study is within the bounds of consent given previously by study members, complies with MRC guidance on ethics and research governance, and meets rigorous MRC data security standards.

## Results

In the maximum sample, mean number of words recalled in the memory test was highest at age 43 and lowest at age 69; mean processing speed was lower at every subsequent age (table 1).

**Table 1:**
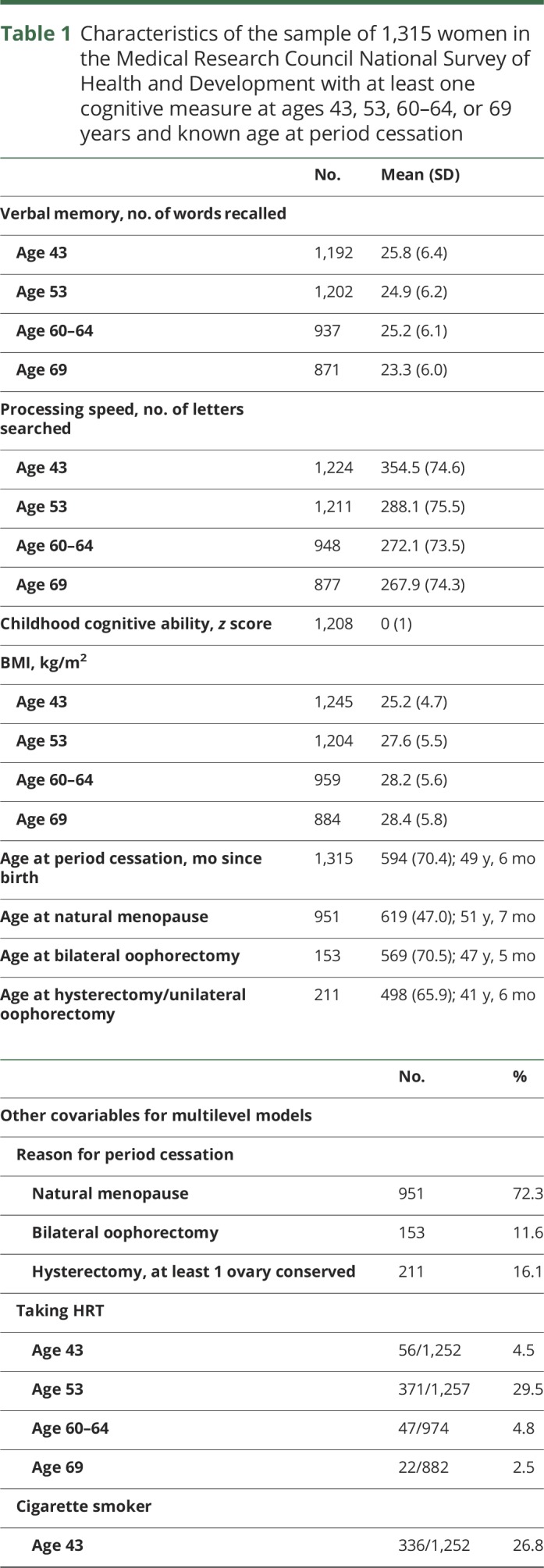

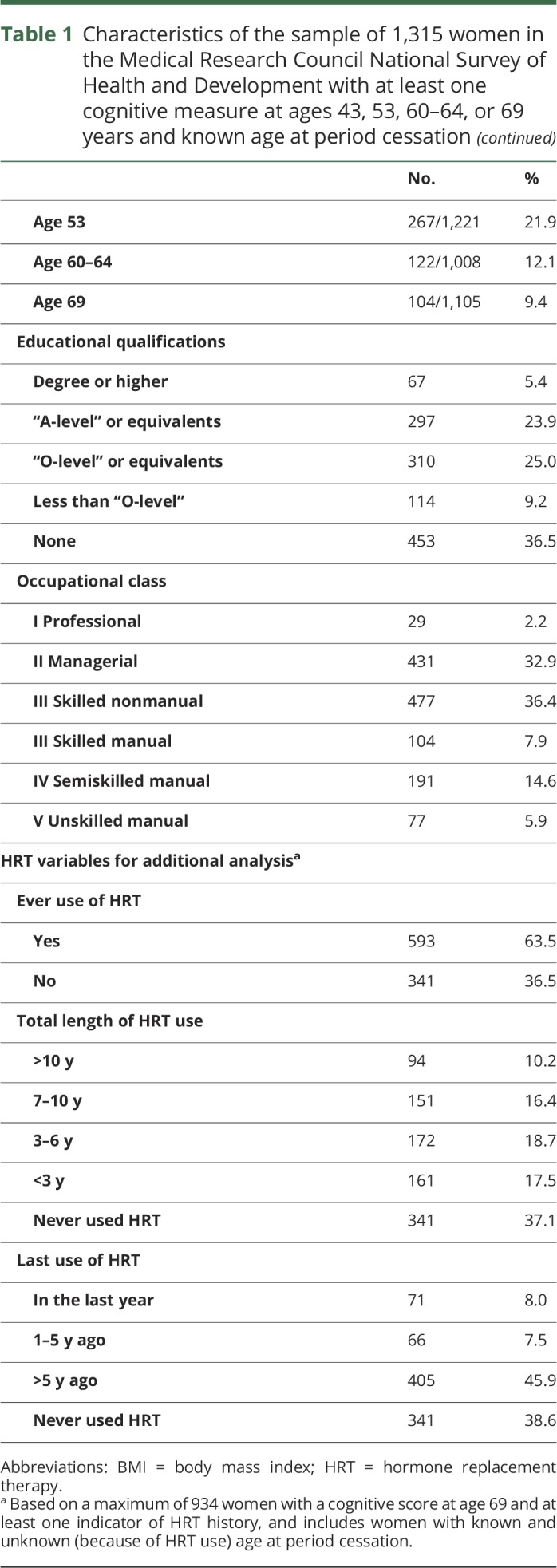
Characteristics of the sample of 1,315 women in the Medical Research Council National Survey of Health and Development with at least one cognitive measure at ages 43, 53, 60–64, or 69 years and known age at period cessation

Mean age at period cessation was 49 years, 7 months: 72% had a natural menopause (mean age 51 years, 7 months), 12% a bilateral oophorectomy (mean age 47 years, 5 months), and 16% a hysterectomy with at least one conserved ovary (mean age 41 years, 6 months) before the natural menopause. HRT use was greatest at age 53 with small numbers of women taking HRT at other ages. Overall, 64% had taken HRT, almost half for 3 or more years, although only 16% had been taking it in the 5 years preceding the last cognitive assessment (table 1). Between ages 43 and 69, mean BMI increased and prevalence of smoking decreased. While the majority of women were in nonmanual occupations, more than a third (36%) had no educational qualifications. Those not providing complete data had similar characteristics to those with complete data. Women lost to follow-up were more likely to have been smokers, had a slightly higher BMI at age 43, and had lower childhood cognitive ability, educational attainment, and occupational class.

### Multilevel models for verbal memory, ages 43–69 years

In 846 women with a natural menopause and complete covariable data, verbal memory increased with later age at natural menopause (table 2A, model 1). There was no evidence of an interaction with age, the association remaining constant across all ages. Adjusting for HRT use (model 2) did not change this estimate; further adjusting for BMI, educational attainment, and occupational class resulted in some attenuation (model 3) as did further adjustment for childhood cognitive ability (model 4). However, an association between later age at menopause and a higher verbal memory score remained after full adjustment (model 4).

**Table 2:**
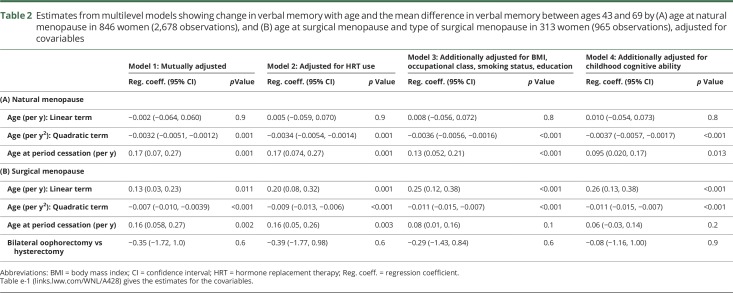
Estimates from multilevel models showing change in verbal memory with age and the mean difference in verbal memory between ages 43 and 69 by (A) age at natural menopause in 846 women (2,678 observations), and (B) age at surgical menopause and type of surgical menopause in 313 women (965 observations), adjusted for covariables

In 313 women with a hysterectomy or bilateral oophorectomy and complete covariable data, verbal memory increased with later age at surgery (table 2B, model 1). There was no evidence of an interaction with age, no difference in memory by type of procedure, and no interaction between age at surgery and type of procedure. Adjusting for HRT use (model 2) did not change the estimate for age at period cessation; there was no interaction between HRT use and type of procedure. Further adjusting for BMI, educational attainment, and occupational class more than halved this estimate (model 3), and after adjusting for childhood cognitive ability, no association remained (model 4).

Childhood cognitive ability, educational attainment, and occupational class were the covariables with the strongest associations with verbal memory (table e-1, links.lww.com/WNL/A428). HRT use at the time of cognitive assessment was not associated with verbal memory for women with a natural menopause; however, for women with surgical menopause, HRT use was associated with a lower verbal memory score and this inverse association remained in the fully adjusted models.

### Multilevel models for processing speed, ages 43–69 years

In 847 women with a natural menopause and complete covariable data, processing speed was not associated with age at natural menopause in any of the models (table 3A, models 1–4). In the fully adjusted model, there was an inverse association with BMI and an association with childhood cognitive ability that increased with age (table e-2, links.lww.com/WNL/A428, model 2). In 314 women with a hysterectomy or bilateral oophorectomy and complete covariable data, neither age at surgical procedure nor any of the covariables were associated with processing speed (table 3B, table e-2).

**Table 3:**
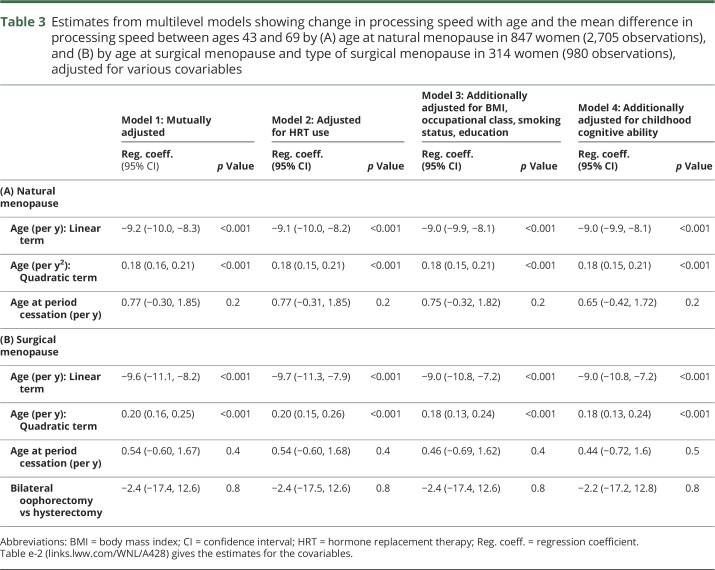
Estimates from multilevel models showing change in processing speed with age and the mean difference in processing speed between ages 43 and 69 by (A) age at natural menopause in 847 women (2,705 observations), and (B) by age at surgical menopause and type of surgical menopause in 314 women (980 observations), adjusted for various covariables

### Additional analyses

The results at each age were consistent with results of the multilevel models (tables e-3 and e-4, links.lww.com/WNL/A428). The results from the multilevel models were essentially unchanged after (1) excluding 28 women who underwent surgery for cancer (24 had surgical menopause and 4 had surgery after a natural menopause), (2) excluding 48 women who had surgical menopause after age 50, and (3) additionally adjusting for parity. There was no evidence that ever using HRT, length of use, or years since last use were associated with verbal memory at age 69. For processing speed, there was some evidence that HRT use was associated with faster processing speed in women who had a bilateral oophorectomy (table e-5).

## Discussion

In a large, national population-based British birth cohort, women who had a later natural menopause maintained a small benefit in verbal memory but not processing speed through to age 69 compared with women with earlier menopause. This benefit was not seen for women who had a surgical menopause after adjustments, or for HRT users. Our longitudinal findings suggest that the association between timing of natural menopause and cognition is not just transient^22^ but is present throughout midlife and persists into the seventh decade. This supports the idea that the relationship between age at period cessation and verbal memory is driven by lifelong processes (such as lifetime estrogen exposure) rather than short-term hormonal fluctuations at the menopause transition.

Our long length of follow-up complements the US SWAN (Study of Women’s Health Across the Nation) study, established to address many of the limitations of previous studies, which showed transient cognitive decrements in perimenopause that did not remain post menopause.^22^ Our study supports and extends previous studies^1^ by having repeat cognitive assessments and by adjusting not only for education (which many of the studies have done) but also adult occupational class and childhood cognition, which were each shown to be independently associated with verbal memory. Only one other study has adjusted for childhood cognitive ability^6^ and found no association between timing of menopause and general cognitive function in a Scottish birth cohort at age 65.

We found age at natural menopause was associated with verbal memory but not processing speed. This differential effect is consistent with evidence from a variety of neurobiological studies. Mechanisms are likely to involve estrogen receptor β function; for example, this regulates brain-derived neurotrophic factor, which in turn solidifies memory formation and storage among other neuroprotective functions. Brain-derived neurotrophic factor has a particularly high concentration in the hippocampus, a subcortical brain structure important for memory.^23^

Despite similar unadjusted associations of ages at natural and surgical menopause with verbal memory, the latter association was explained by other factors. Previous studies have not always distinguished between type of menopause,^1^ or type of and reasons for surgery,^21^ and results have been inconsistent. However, Bove et al.^24^ showed in 2 longitudinal studies that earlier age at surgical menopause was associated with faster decline in global cognition and increased Alzheimer disease neuropathology, controlling only for age, education, smoking, and cohort. Other studies have shown that women who have a surgical menopause have faster cognitive decline than those who have a natural menopause. For example, in an analysis of 3 trials, bilateral oophorectomy was associated with decline in visual memory over 2.7 years in women having the procedure after age 45, and with decline in semantic memory for women having the procedure before age 45, compared with natural menopause.^25^ In our study, we found small differences in verbal memory by type of menopause.

The effects of HRT use on cognition or dementia risk were originally thought to be positive,^26^ until the results of the Women's Health Initiative showed adverse effects in older women.^27,28^ The possibility that HRT could be beneficial during a “critical window” at the time of the menopause transition^29,30^ led to a follow-up study^31^ and recent trials in younger women^32,33^ that showed no such beneficial effects on cognition. Our study showed that, for most women, there was no evidence that HRT use promoted better cognitive performance—indeed, estimates were generally negative. Our additional analyses showed HRT use was only protective for processing speed in women with a bilateral oophorectomy. HRT use decreased in this cohort during 2002 (age 56) at the time of adverse trial reports,^34^ and later-born cohorts have been less likely to use HRT. Therefore, our HRT results may not be generalizable to later-born cohorts. Given the decrease in HRT use, HRT may have only been prescribed to women at probable high risk of adverse health conditions thought to be associated with early period cessation. This could have weakened any positive associations between HRT use and cognition. However, our findings are consistent with the recent trials in younger women^32,33^ and with the Women's Health Initiative/Women's Health Initiative Memory Study, which showed an increase in dementia cases in women aged 65 to 79 years in the combined and estrogen-only arms of the trial.^27,28^ Findings from the Women's Health Initiative Memory Study–MRI were consistent in showing that women receiving HRT had significant losses of gray matter compared with the placebo groups.^35^

The relatively large gaps between cognitive assessments in our study are a limitation. Those lost to follow-up (but not those with missing data) had less favorable childhood cognitive ability and sociobehavioral characteristics, and their exclusion may have led to weaker associations between menopause timing and adult cognition. HRT dose was not collected and data on types of HRT preparations were insufficiently complete to use. However, the vast majority receiving HRT who had undergone a hysterectomy or oophorectomy were taking estrogen alone, whereas other women were taking a combined preparation.^36^ Data on length of use, age at last use, and whether HRT use was initiated close to period cessation were advantages over studies that only collected measures of current and past HRT use.

The oldest of the British birth cohort studies, NSHD is one of the few with sufficiently long-term follow-up to investigate common early-life factors associated with lifelong reproductive and cognitive function. Key strengths of our study are its relatively large sample of women of the same age, prospective data on type of menopause, age at period cessation and HRT, childhood cognitive ability, repeat measures of fluid cognition across adult life, and the approximately 15 years of follow-up since menopause for most women.

The HRT findings are of relevance to National Institute for Health and Care Excellence recommendations for good quality observational studies controlling for the effect of important confounders on how early HRT use affects dementia risk in women with early menopause.^37^ Overall, the implications of our findings are that modifiable factors that delay reproductive aging, in addition to those that promote cognitive development, may have small beneficial effects on later-life cognition. While this finding is of etiologic relevance, the benefits are small: the difference in verbal memory scores in the fully adjusted models for a 10-year difference in age at natural menopause was one word, equivalent to a sixth of a standard deviation on that score. Continued follow-up will determine whether this benefit to memory eventually translates into reduced dementia risk.

### Data sharing

Data used in this publication are available to bona fide researchers on request to the NSHD Data Sharing Committee via a standard application procedure. Further details can be found at nshd.mrc.ac.uk/data. Doi: 10.5522/NSHD/Q102; 10.5522/NSHD/Q103.

## Glossary

BMI: body mass index
CI: confidence interval
HRT: hormone replacement therapy
MRC: Medical Research Council
NSHD: National Survey of Health and Development

## References

[1] Georgakis MK, Kalogirou EI, Diamantaras AA, et al. Age at menopause and duration of reproductive period in association with dementia and cognitive function: a systematic review and meta-analysis. Psychoneuroendocrinology 2016;73:224–243. 27543884 10.1016/j.psyneuen.2016.08.003

[2] Henderson VW. Gonadal hormones and cognitive aging: a midlife perspective. Womens Health 2011;7:81–93. 10.2217/whe.10.87 PMC3675221 21175393

[3] Richards M, Kuh DL, Hardy R, Wadsworth MEJ. Lifetime cognitive function and timing of natural menopause. Neurology 1999;53:308–314. 10430419 10.1212/wnl.53.2.308

[4] Kuh D, Butterworth S, Kok H, et al. Childhood cognitive ability and age at menopause: evidence from two cohort studies. Menopause 2005;12:475–482. 16037764 10.1097/01.GME.0000153889.40119.4C

[5] Mishra G, Hardy R, Kuh D. Are the effects of risk factors for timing of menopause modified by age? Results from a British birth cohort study. Menopause 2007;14:717–724. 17279060 10.1097/GME.0b013e31802f3156

[6] Whalley LJ, Fox HC, Starr JM, Deary IJ. Age at natural menopause and cognition. Maturitas 2004;15:148–156. 10.1016/j.maturitas.2003.12.014 15474759

[7] Cooper R, Hardy R, Kuh D. Timing of menarche, childbearing and hysterectomy risk. Maturitas 2008;614:317–322. 10.1016/j.maturitas.2008.09.025 PMC3500690 19013032

[8] Richards M, Shipley B, Fuhrer R, Wadsworth ME. Cognitive ability in childhood and cognitive decline in mid-life: longitudinal birth cohort study. Br Med J 2004;328:552–554. 14761906 10.1136/bmj.37972.513819.EE PMC381045

[9] Gow AJ, Johnson W, Pattie A, et al. Stability and change in intelligence from age 11 to ages 70, 79, and 87: the Lothian Birth Cohorts of 1921 and 1936. Psychol Aging 2011;26:232–240. 20973608 10.1037/a0021072

[10] Kok HS, Kuh D, Cooper R, et al. Cognitive function across the life course and the menopausal transition in a British birth cohort. Menopause 2006;13:19–27. 16607095 10.1097/01.gme.0000196592.36711.a0

[11] Koebele SV, Bimonte-Nelson HA. Trajectories and phenotypes with estrogen exposures across the lifespan: what does Goldilocks have to do with it? Horm Behav 2015;74:86–104. 26122297 10.1016/j.yhbeh.2015.06.009 PMC4829405

[12] Hara Y, Waters EM, McEwen BS, Morrison JH. Estrogen effects on cognitive and synaptic health over the lifecourse. Physiol Rev 2015;95:785–807. 26109339 10.1152/physrev.00036.2014 PMC4491541

[13] Au A, Feher A, McPhee L, Jessa A, Oh S, Einstein G. Estrogens, inflammation and cognition. Front Neuroendocrinol 2016;40:87–100. 26774208 10.1016/j.yfrne.2016.01.002

[14] Kuh D, Wong A, Shah I, et al. The MRC National Survey of Health and Development reaches age 70: maintaining participation at older ages in a birth cohort study. Eur J Epidemiol 2016;31:1135–1147. 27995394 10.1007/s10654-016-0217-8 PMC5206260

[15] Richards M, Sacker A. Lifetime antecedents of cognitive reserve. J Clin Exp Neuropsychol 2003;25:614–624. 12815499 10.1076/jcen.25.5.614.14581

[16] Albanese E, Hardy R, Wills A, Kuh D, Guralnik J, Richards M. No association between gain in body mass index across the life course and midlife cognitive function and cognitive reserve: the 1946 British Birth Cohort study. Alzheimers Dement 2012;8:470–482. 22858531 10.1016/j.jalz.2011.09.228 PMC3778923

[17] Richards M, Jarvis M, Thompson N, Wadsworth M. Cigarette smoking and cognitive decline in midlife: longitudinal population based study. Am J Public Health 2003;93:994–998. 12773367 10.2105/ajph.93.6.994 PMC1447882

[18] Cooper R, Hardy R, Kuh D. Is adiposity across life associated with subsequent hysterectomy risk? Findings from the 1946 British birth cohort study. BJOG 2008;115:184–192. 18081600 10.1111/j.1471-0528.2007.01569.x

[19] Hardy R, Kuh D, Wadsworth M. Smoking, body mass index, socioeconomic status and the age at menopause transition in a British national cohort. Int J Epidemiol 2000;29:845–851. 11034967 10.1093/ije/29.5.845

[20] Hardy R, Kuh D. Social and environmental conditions across the life course and age at menopause in a British birth cohort study. BJOG 2005;112:346–354. 15713152 10.1111/j.1471-0528.2004.00348.x

[21] Hogervorst E. Oophorectomy and hysterectomy may increase dementia risk but only when performed prematurely. J Alzheimers Dis 2014;42:583–586. 24898655 10.3233/JAD-140909

[22] Greendale GA, Huang MH, Wight RG, et al. Effects of the menopause transition and hormone use on cognitive performance in midlife women. Neurology 2009;72:1850–1857. 19470968 10.1212/WNL.0b013e3181a71193 PMC2690984

[23] Zhao L, Woody SK, Chhibber A. Estrogen receptor beta in Alzheimer's disease: from mechanisms to therapeutics. Ageing Res Rev 2015;24:178–190. 26307455 10.1016/j.arr.2015.08.001 PMC4661108

[24] Bove R, Secor E, Chibnik LB, et al. Age at surgical menopause influences cognitive decline and Alzheimer pathology in older women. Neurology 2014;82:222–229. 24336141 10.1212/WNL.0000000000000033 PMC3902759

[25] Kurita K, Henderson VW, Gatz M, et al. Association of bilateral oophorectomy with cognitive function in healthy, postmenopausal women. Fertil Steril 2016;106:749–756. 27183047 10.1016/j.fertnstert.2016.04.033 PMC5743432

[26] LeBlanc ES, Janowsky J, Chan BK, Nelson HD. Hormone replacement therapy and cognition: systematic review and meta-analysis. JAMA 2001;285:1489–1499. 11255426 10.1001/jama.285.11.1489

[27] Shumaker SA, Legault C, Kuller L, et al. Conjugated equine estrogens and incidence of probable dementia and mild cognitive impairment in postmenopausal women: Women's Health Initiative Memory Study. JAMA 2004;291:2947–2958. 15213206 10.1001/jama.291.24.2947

[28] Shumaker SA, Legault C, Rapp SR, et al. Estrogen plus progestin and the incidence of dementia and mild cognitive impairment in postmenopausal women. JAMA 2003;289:2651–2662. 12771112 10.1001/jama.289.20.2651

[29] Maki PM. Critical window hypothesis of hormone therapy and cognition: a scientific update on clinical studies. Menopause 2013;20:695–709. 23715379 10.1097/GME.0b013e3182960cf8 PMC3780981

[30] McCarrey AC, Resnick SM. Postmenopausal hormone therapy and cognition. Horm Behav 2015;74:167–172. 25935728 10.1016/j.yhbeh.2015.04.018 PMC4573348

[31] Espeland MA, Shumaker SA, Leng I, et al. Long-term effects on cognitive function of postmenopausal hormone therapy prescribed to women aged 50 to 55 years. JAMA Intern Med 2013;173:1429–1436. 23797469 10.1001/jamainternmed.2013.7727 PMC3844547

[32] Henderson VW, St John JA, Hodis HN, et al. Cognitive effects of estradiol after menopause: a randomized trial of the timing hypothesis. Neurology 2016;87:699–708. 27421538 10.1212/WNL.0000000000002980 PMC4999165

[33] Gleason CE, Dowling NM, Wharton W, et al. Effects of hormone therapy on cognition and mood in recently postmenopausal women: findings from the randomized, controlled KEEPS-Cognitive and Affective Study. PLoS Med 2015;12:e1001833. 26035291 10.1371/journal.pmed.1001833 PMC4452757

[34] Mishra G, Kok H, Ecob R, Cooper R, Hardy R, Kuh D. Cessation of hormone replacement therapy after reports of adverse findings from randomized controlled trials: evidence from a British birth cohort. Am J Public Health 2006;96:1219–1225. 16735620 10.2105/AJPH.2005.071332 PMC1483866

[35] Zhang T, Casanova R, Resnick SM, et al. Effects of hormone therapy on brain volumes changes of postmenopausal women revealed by optimally-discriminative voxel-based morphometry. PLoS One 2016;11:e0150834. 26974440 10.1371/journal.pone.0150834 PMC4790922

[36] Kuh D, Hardy R, Wadsworth M. Social and behavioural influences on the uptake of hormone replacement therapy among younger women. Br J Obstet Gynaecol 2000;107:731–739. 10.1111/j.1471-0528.2000.tb13333.x 10847228

[37] National Institute for Health and Care Excellence. Menopause: Diagnosis and Management: NICE Guideline. London: National Institute for Health and Care Excellence; 2015.

